# The association between telomere length and ischemic stroke risk and phenotype

**DOI:** 10.1038/s41598-021-90435-9

**Published:** 2021-05-26

**Authors:** Ezgi Yetim, Mehmet Akif Topcuoglu, Nuket Yurur Kutlay, Ajlan Tukun, Kader K. Oguz, Ethem Murat Arsava

**Affiliations:** 1 Department of Neurology, Faculty of Medicine, Hacettepe University, Sihhiye, 06100 Ankara, Turkey; 2 Department of Medical Genetics, Faculty of Medicine, Ankara University, Ankara, Turkey; 3 Department of Medical Genetics, Duzen Laboratory Group, Ankara, Turkey; 4 Department of Radiology, Faculty of Medicine, Hacettepe University, Ankara, Turkey

**Keywords:** Stroke, Molecular medicine

## Abstract

The chronological age of a person is a key determinant of etiology and prognosis in the setting of ischemic stroke. Telomere length, an indicator of biological aging, progressively shortens with every cell cycle. Herein, we determined telomere length from peripheral blood leukocytes by Southern blot analyses in a prospective cohort of ischemic stroke patients (n = 163) and equal number of non-stroke controls and evaluated its association with various ischemic stroke features including etiology, severity, and outcome. A shorter telomere length (i.e. lowest quartile; ≤ 5.5 kb) was significantly associated with ischemic stroke (OR 2.95, 95% CI 1.70–5.13). This significant relationship persisted for all stroke etiologies, except for other rare causes of stroke. No significant association was present between admission lesion volume and telomere length; however, patients with shorter telomeres had higher admission National Institutes of Health Stroke Scale scores when adjusted for chronological age, risk factors, etiology, and infarct volume (p = 0.046). On the other hand, chronological age, but not telomere length, was associated with unfavorable outcome (modified Rankin scale > 2) and mortality at 90 days follow-up. The association between shorter telomere length and more severe clinical phenotype at the time of admission, might reflect reduced resilience of cerebral tissue to ischemia as part of biological aging.

## Introduction

The severity and course of functional impairment are highly heterogeneous among patients with ischemic stroke^1,2^. The major and indisputable determinants of stroke severity include the size and location of the ischemic territory, which is inherently related to the site of arterial occlusion^3–5^. On the other hand, a multitude of patient related factors, such as age, gender, admission glucose level, baseline vascular risk factors, cognitive status, and small vessel disease burden have been shown to alter the fate of tissue and clinical outcome, and thereby significantly contribute to the above-mentioned heterogeneity in terms of outcome^6–12^. Although the underlying pathophysiology affecting the prognosis is highly different for each of these factors, they can overall be considered to reflect the resilience of cerebral tissue to ischemic injury.

Among these individual factors, the most robust and widely-studied one is age. Both experimental stroke models and human studies have consistently shown that young age increases resistance to ischemia and contributes positively to recovery after ischemic injury^6,11,13–15^. In this regard, functional outcome, when adjusted for baseline stroke severity, is better in young individuals with ischemic stroke, while stroke-related mortality and morbidity are higher in the older age group^6,11,14^. In addition to adverse results in terms of clinical outcome, aging is also an unfavorable prognostic factor for tissue outcome; the conversion of ischemic but viable tissue to infarction within the penumbra region and the overall ischemic lesion growth is more common in the elderly population^16^.

Despite the extensive literature highlighting the importance of age in various aspects of stroke prognosis, the correlation is far from perfect. One reason, apart from the role of other individual prognostic factors, is the fact that chronological age, as used in almost all of these studies, does not reflect biological age in a homogenous fashion^17,18^. This discrepancy in terms of chronological and biological aging in the human body, which of course is not only important from the perspective of cerebrovascular diseases, has ignited research on identifying certain genetic or biochemical biomarkers that might be helpful in more accurately evaluating the biological age of the organism. One traditionally used tool in this regard are telomeres, the repetitive DNA sequences located at the end of chromosomes, whose length progressively shorten with every cell cycle. The shortening of telomere length has therefore been considered a reflection of the biological clock of the aging organism^19,20^. Prior reports have shown the presence of shorter telomeres in patients with cardiovascular disease and stroke^21–24^. However, the contribution of telomere length to stroke severity and prognosis has not been evaluated sufficiently. In this study, our aim was to evaluate the association between telomere length and ischemic stroke, not only in terms of stroke risk in general but also from the perspective of stroke etiology and severity.

## Results

Our study population was comprised of 163 ischemic stroke patients and an equal number of non-stroke controls. The baseline characteristics of the study population are summarized in Table 1. Ischemic stroke patients, expectedly, had higher rates of vascular risk factors, except for active smoking. The median (interquartile range, IQR) telomere length was 7.0 (5.5–9.0) kb and was negatively correlated with age (r = − 0.23; p < 0.001) in the overall study population. However, this negative correlation was primarily driven by the ischemic stroke cohort (Supplemental Fig. I). The median (IQR) telomere length was non-significantly shorter in patients with ischemic stroke patients in comparison to controls [7 (5–9) kb vs. 7 (6–9) kb; p = 0.095].

**Table 1:** Baseline characteristics of the study population.

	Ischemic Stroke (n = 163)	Non-stroke controls (n = 163)	p
Age	69 (58–78) years	68 (61–73) years	0.444
Female gender	80 (49%)	81 (50%)	0.912
Hypertension	123 (76%)	99 (61%)	0.004
Diabetes Mellitus	60 (37%)	37 (23%)	0.005
Coronary artery disease	53 (33%)	42 (26%)	0.180
Hyperlipidemia	93 (57%)	82 (50%)	0.222
Active smoking	35 (22%)	45 (28%)	0.198

**Figure 1:**
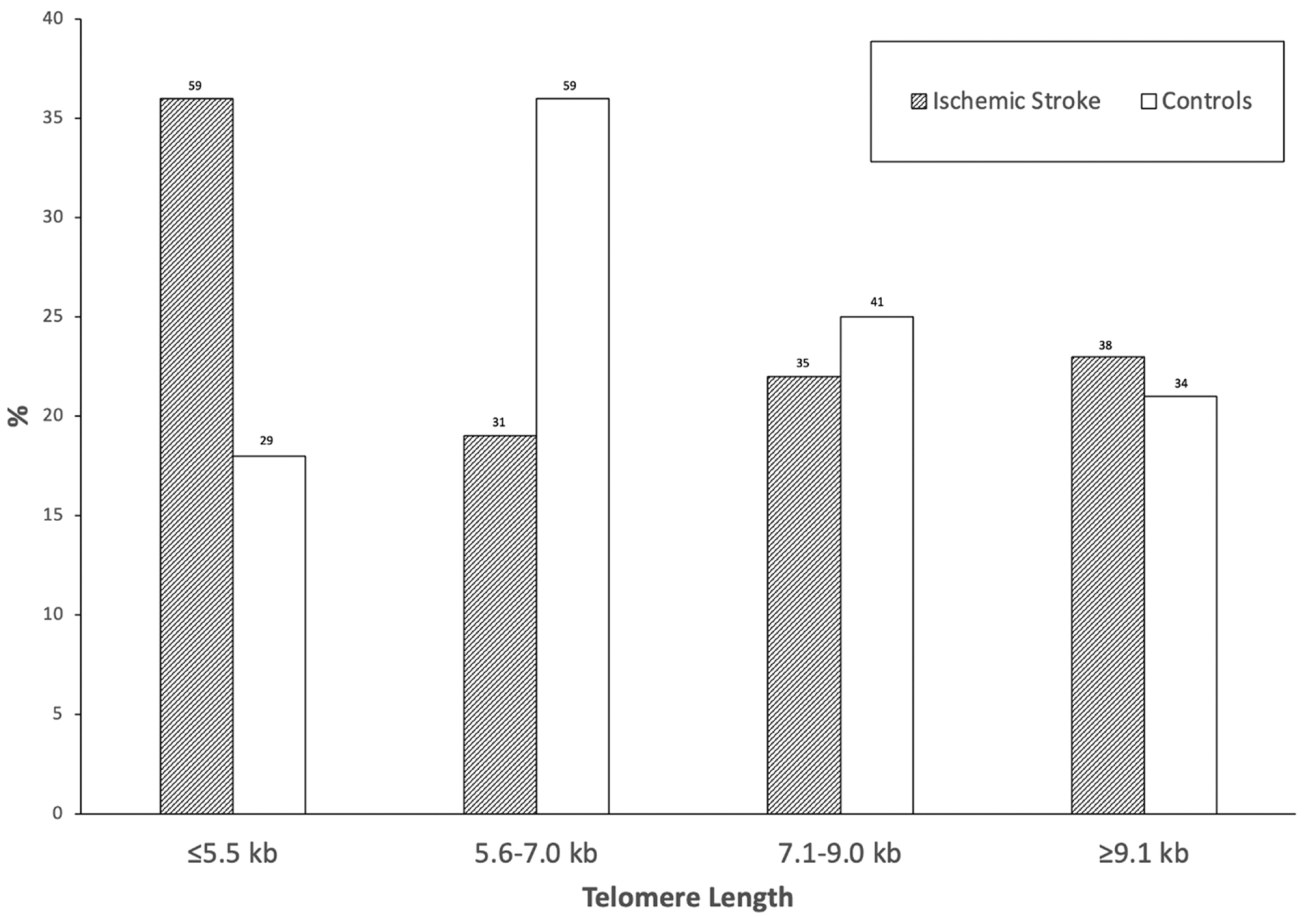
The distribution of telomere length in quartiles among ischemic stroke patients and controls. The numbers above bars represent the actual number of observed cases in each category.

Figure 1 shows the distribution of telomere length in quartiles among ischemic stroke patients and controls. Ischemic stroke patients were more likely to harbor shorter telomeres (i.e. lowest quartile; ≤ 5.5 kb) in comparison to controls (36% vs. 18%; p < 0.001). Among subjects with short telomere length, stroke patients were more likely to be older than controls [77 (67–84) vs. 69 (64–77) years; p = 0.047]. The significant association between shorter telomere length and ischemic stroke persisted when adjusted for age, gender, and baseline risk factors [OR 2.95 (95% CI 1.70–5.13); p < 0.001]. The underlying stroke etiology, as evaluated by the Causative Classification of Stroke (CCS) system, was large artery atherosclerosis in 29 (18%) of our stroke patients, cardio-aortic embolism in 53 (33%), small artery occlusion in 11 (7%), other causes in 6 (4%), and undetermined in 64 (39%). The distribution of telomere length was not significantly different among various stroke subtypes (p = 0.243). A link between short telomere length and ischemic stroke was observed, similar to the comparison of overall stroke cohort to controls, when analyses were repeated for separate etiologic subgroups, except for the ‘other causes’ etiology of which none of the ischemic stroke patients had a telomere length of ≤ 5.5 kb (Supplementary Table S1).

Table 2 summarizes the clinical features of the stroke cohort stratified according to telomere length. Ischemic stroke patients with shorter telomeres were more likely to be older (p < 0.001), female (p = 0.049), and have a history of hypertension (p = 0.038) and coronary artery disease (p = 0.018) in univariate analyses. The admission diffusion-weighted imaging (DWI) lesion volume was not different between the groups; however, the admission National Institutes of Health Stroke Scale (NIHSS) score was significantly higher in ischemic stroke patients with a telomere length of ≤ 5.5 kb (p = 0.039). Accordingly, patients within the shortest telomere length quartile were less likely to have a favorable neurological outcome (p = 0.016) and more likely to die (p = 0.0018) at 3 months.

**Table 2:** Clinical features of ischemic stroke patients stratified according to telomere length.

	Telomere length		p
	≤ 5.5 kb (n = 59)	> 5.5 kb (n = 104)	
Age	77 (67–84)	67 (52–74)	< 0.001
Female gender	35 (59%)	45 (43%)	0.049
Hypertension	50 (85%)	73 (70%)	0.038
Diabetes Mellitus	26 (44%)	34 (33%)	0.148
Coronary artery disease	26 (44%)	27 (26%)	0.018
Hyperlipidemia	32 (54%)	61 (59%)	0.584
Active smoking	10 (17%)	25 (24%)	0.289
Admission NIHSS score	6 (3–17)	4 (2–9)	0.039
Admission DWI lesion volume (mL)	6.0 (1.1–30.7)	6.4 (0.9–28.1)	0.585
CCS stroke subtype			0.221
Large artery atherosclerosis	11 (19%)	18 (17%)	
Cardio-aortic embolism	23 (39%)	30 (29%)	
Small artery occlusion	5 (9%)	6 (6%)	
Other causes	0 (0%)	6 (6%)	
Undetermined	20 (34%)	44 (42%)	
90-day mRS ≤ 2	26 (44%)	66 (63%)	0.016
Death	12 (20%)	8 (8%)	0.018

The association between short telomere length and female gender, history of hypertension, and coronary artery disease became non-significant when adjusted for age among stroke patients. However, shorter telomere length remained positively associated with admission NIHSS score, together with admission DWI lesion volume, history of hypertension, and cardio-aortic embolism after adjustment for patient age in multivariate analysis (Table 3). When the analyses were repeated after categorizing stroke severity into severe (admission NIHSS ≥ 16; n = 31) and non-severe strokes, a shorter telomere length was significantly associated with a severe stroke phenotype [OR: 5.95 (1.58–22.36); p = 0.008]. The results of the analyses did not change when infarct location (categorized as left hemisphere, right hemisphere, posterior circulation) was introduced into the model as a covariate. Chronological age was not associated with stroke severity in either of these models. Neither patient age, nor telomere length was related to ischemic lesion volume on admission DWI; stroke etiology was the only significant parameter associated with lesion size in multivariate analyses, with larger lesions in patients with cardio-aortic embolism and smaller lesions in patients with small artery occlusion. On the other hand, older chronological age and higher admission NIHSS score were the only significant variables associated with unfavorable 90-day functional outcome (modified Rankin Scale, mRS > 2) and mortality.

**Table 3:** Multivariate predictors of admission stroke severity.

Model 1: Dependent variable: admission NIHSS score		
	Beta coefficient	p
Admission DWI lesion volume	0.010	< 0.001
Hypertension	0.364	0.035
Telomere length ≤ 5.5 kb	0.298	0.046
**CCS stroke subtype**		
Large artery atherosclerosis	− 0.199	0.843
Cardio-aortic embolism	1.843	0.067
Small artery occlusion	− 0.548	0.585
Other causes	− 1.203	0.231
Undetermined	Reference	

Model 2: Dependent variable: admission NIHSS score (< 16 vs. ≥ 16)		
	OR (95% CI)	p
Admission DWI lesion volume	1.07 (1.04–1.09)	< 0.001
Coronary artery disease	5.90 (1.62–21.48)	0.007
Hyperlipidemia	0.33 (0.09–1.15)	0.082
Telomere length ≤ 5.5 kb	5.95 (1.58–22.36)	0.008

## Discussion

Our findings replicate previous studies in the literature showing the presence of a shorter telomere length in patients suffering from ischemic stroke^21–26^. We extend this observation by highlighting that a similar relationship exists for major ischemic stroke etiologies, except for those secondary to other rare causes. In addition, we documented a more severe ischemic stroke phenotype among those patients with a shorter telomere length, when adjusted for confounding variables including lesion volume and stroke etiology. Importantly such an effect on stroke severity was not found for the chronological age of the patient, which was on the other hand more important from the perspective of long-term functional outcome and mortality after stroke.

The association between shorter telomere length with cardiovascular disease, and specifically with stroke has been the subject of several epidemiologic studies^21,23,25^. Recent systematic reviews and meta-analyses reported a consistent relationship of decreased leukocyte telomere length with cardiometabolic outcomes, with at least more than ~ 20% increased stroke prevalence among those with short telomeres^23,25^. The cellular senescence involving the cardiac and cerebral vasculature is considered to drive these associations related to cardiovascular diseases. An increased cell turnover triggered by shear stress, oxidative stress, and inflammation has been put forward to underlie the mutual interplay between atherosclerosis and shorter telomeres^27^. On the other hand, mechanisms underlying the association between telomere length and other subtypes of strokes, such as lacunes, cardioembolism, or hemorrhagic stroke are less well studied and understood^22,24,26^. Evaluation of the role of telomere length in specific ischemic stroke etiologies was one of the major goals of our study. The identification of a shorter telomere length was approximately three or four times more common in patients with stroke secondary to large artery atherosclerosis, cardio-aortic embolism, and undetermined etiology, in comparison to the control population. The association was more robust in the small artery occlusion group (with an odds ratio of ~ 5), however, the low number of patients in this group necessitates validation of this observation in other cohorts. Overall, these findings suggest that a shorter telomere length is associated with most ischemic stroke etiologies, except for other rare causes, possibly as a reflection of vascular aging in general. However, some yet undiscovered aspects of aging that contribute to specific stroke etiologies might fine-tune the degree of this relationship.

Certain patient-related factors alter the stroke phenotype in terms of admission stroke severity, even when adjusted for its primary determinants including ischemic lesion volume, topographic distribution, and stroke etiology^3,5,14^. Despite the well-established role of advanced age in terms of unfavorable functional outcome in the long-term^5,6,11,13^, there are controversial reports regarding the effects of aging on symptom severity at the time of admission. Certain publications have highlighted the presence of more severe neurological deficits in the elderly population^28,29^; yet, as these associations were significantly weakened when confounders were taken into account, age is usually not considered a key predictor of symptom severity^30^. On the other hand, certain aging-related conditions like leukoaraiosis burden, baseline dementia, or pre-stroke frailty show a more robust association with admission NIHSS scores^31–33^, all of which are well-known to be related to shorter telomere length^34–36^. Overall, these observations corroborate our findings of higher NIHSS scores among ischemic stroke patients with shorter telomeres and suggest that telomere length can be considered as a composite biomarker of aging-related pathological processes involving the brain, which ultimately decrease the resilience of brain tissue to ischemic insults.

In contrast to the significant relationship between telomere length and acute symptom severity, our analyses have revealed chronological age as the key predictor of 90-day outcome. Although there are a multitude of studies that evaluated the relationship between telomere length and stroke^23^, few studies in the literature have focused on the effect on stroke outcome, and highlighted an increased risk of mortality or dementia over the years following a stroke in patients with shorter telomere length^22,26,37^. As for the outcome in a shorter time frame, to the best of our knowledge, there are only studies that made use of epigenetic markers of biological aging rather than telomere length in the stroke population. Contrary to our observations, these studies have suggested that changes in age-related DNA methylation are better predictors of 90-day functional outcome and mortality, in comparison to chronological age^17,18^. Discrepancies among epigenetic (e.g. DNA methylation changes) and mitotic (e.g. telomere length) markers of biological aging are a well-known phenomenon and are considered to indicate different aspects of cellular senescence^38^. In this context, it might be speculated that epigenetic markers of aging might play a more crucial role in terms of the potential for stroke recovery, while telomere length reflects the vulnerability to the initial ischemic insult. Furthermore, any potential effect of telomere length on functional outcome might be hard to capture, due to the significant relationship between telomere length and admission NIHSS score, which itself is a major determinant of 90-day outcome. Finally, the social aspects of chronological age, such as shortage of caregiver support or issues with accessing medical care and rehabilitation, might be more critical than the biological age of the patient in post-stroke recovery period.

Certain limitations of our manuscript merit consideration. We have used telomere length in peripheral blood leukocytes as a marker of biological aging. However, recent studies primarily performed in newborns and children have challenged the validity of telomere length as a biomarker of mitotic clock in humans^39^. Therefore, it is possible that other mechanisms than aging itself might be the main contributors of the associations observed between short telomere length, chronic disease and oxidative stress/inflammation burden in the body, vascular aging and age-related changes in the brain. As another limitation, topographic information of ischemic areas was defined just by gross hemispheric localization and therefore might have underestimated the influence of lesion location in our analyses focusing on stroke severity. We also had a small number of patients with certain stroke etiologies (small artery occlusion, other causes) that hindered us to establish firm conclusions regarding the interplay between some stroke subtypes and telomere length. Finally, despite using a gold-standard approach for measuring telomere length, a major limitation of our study is the lack of duplicate assessments, which precludes us from calculating the intra-assay coefficient of variation in our measurements.

In conclusion, we have not only verified the association between ischemic stroke and shorter telomere length but also have found that telomere length might be related to stroke phenotype primarily in the acute stroke setting. Nonetheless, chronological age was a more robust predictor of 90-day outcome when compared to telomere length. The combination of telomere length analyses with epigenetic markers of aging in future studies, and evaluation of cellular processes that accompany shorter telomere length, together with systemic and neural substrates that contribute to this shortening would help us better understand the pathophysiological role of biological aging in stroke severity and recovery.

## Methods

We prospectively enrolled ischemic stroke patients admitted to our center between the period of November 2013 and July 2015. Only patients admitted within 48 h after symptom onset and had no contraindications to undergo magnetic resonance imaging (MRI) were included in the study. During the same period, participants without any established diagnosis for a neurologic disease or stroke were invited by printed and electronic flyers to serve as the control group of the study. Their medical history was thoroughly reviewed, and those with a diagnosis of cancer, chronic liver, or renal disease were not enrolled. All qualifying controls have also undergone an MRI study, and those with silent territorial strokes, despite the absence of clinical history, were excluded from the study. Among > 350 participants who served as a general control group for our multiple studies focusing on the vascular pathophysiology of stroke, an equal number of subjects with similar age and gender distribution characteristics were selected. A written consent was obtained from study participants or their relatives. Approval was obtained from the ethics committee of Hacettepe University (REC number: GO 13/243). The procedures used in this study adhere to the tenets of the Declaration of Helsinki.

Information related to demographic features and vascular risk factors was collected from both cohorts. In addition, stroke related features, including admission stroke severity [as evaluated by National Institutes of Health Stroke Scale (NIHSS) score], etiology [as evaluated by the Causative Classification of Stroke (CCS) system]^40^, and 90-day functional outcome [as evaluated by the modified Rankin Scale (mRS)] was determined in stroke patients. Admission ischemic lesion volume was measured from DWI by semi-automated segmentation using the MRIcro software (University of Nottingham, UK).

We collected 20 ml of venous blood into two EDTA tubes from all participants to determine telomere length in circulating white blood cells. After DNA extraction from blood samples with the MasterPure™ DNA purification Kit (Epicenter), DNA integrity was evaluated by using 1% agarose gel. A minimum of 3 µg of DNA was used for digestion with *Hinf*I and *HpH*I restriction nucleases. For terminal restriction fragment (TRF) length analysis, a 0.5% agarose gel was used to resolve the digested DNA and the gel was run overnight at 2–2.25 V cm^−1^. Before the transfer of DNA to the membrane, gel depurination, denaturation, and neutralization steps were done respectively. For DNA transfer to the membrane, Whatman Turboblotter Rapid Downward Transfer Systems was used. For hybridization, probes were prepared: Telomere probe was labeled at 3′ end with digoxigenin (DIG); probes for 1 kb and λ DNA/*Hind*III ladders were similarly labeled with DIG by using random primed DNA labeling kit. A Cross-linked membrane was put in the hybridization tube with hybridization solution and was rotated approximately 3 days at 37 °C in the hybridization oven. For chemiluminescence detection, the protocol was applied and then telomere length in transparency film was measured according to the ladders^41^. All TRF length analyses were performed while blinded to the entire clinical data.

### Statistical analysis

Categorical variables are expressed as n (%) and continuous variables as median (interquartile range). Group-wise comparisons were performed by chi-square test for categorical variables and Mann–Whitney U test for continuous variables. Logistic regression and linear regression models were constructed to determine independent factors related to stroke severity (admission NIHSS score), admission DWI lesion volume, and functional outcome (90-day mRS and mortality). Dependent variables in linear regression analyses (admission NIHSS score and DWI lesion volume) were log-transformed prior to being introduced into the models in order to comply with the assumption of normality. Among other variables, both patient age and telomere length were introduced into these models as independent variables. A backward selection algorithm with retention criteria of p < 0.10 was used in the multivariate models to prevent overfitting. A p < 0.05 was considered statistically significant. SPSS version 21.0 was used for statistical analyses.

### Informed consent

Written informed consent was obtained from the all participants or their representatives for their anonymized information to be published in this article.

### Ethics approval

This study was approved by the institutional ethics review committee (REC number: GO 13/243).

## Supplementary Information

Supplementary Information.

## Data Availability

The data that support the findings of this study are available from the corresponding author upon reasonable request.
