# Supplementary material for: The association between telomere length and ischemic stroke risk and phenotype
Source: Sci Rep. 2021 May 26;11:10967. doi: 10.1038/s41598-021-90435-9 (PMC8155040; doi:10.1038/s41598-021-90435-9)

**Supplemental Table I:** The association between shorter telomere length (≤5.5 kb) and ischemic stroke subtypes after adjustment for baseline risk factors.

|  | **OR (95% CI)** | **p** |
| --- | --- | --- |
| Large artery atherosclerosis (n=29) | 3.70 (1.38-9.94) | 0.009 |
| Cardio-aortic embolism (n=53) | 3.37 (1.57-7.22) | 0.002 |
| Small artery occlusion (n=11) | 4.96 (1.05-23.33) | 0.043 |
| Other causes (n=6) | Not calculated* | - |
| Undetermined (n=64) | 2.51 (1.26-5.02) | 0.009 |

*all six patients with ischemic stroke due to other causes had a telomere length >5.5 kb

Independent variables in the models: age, gender, hypertension, diabetes mellitus, coronary artery disease, hyperlipidemia, active smoking, telomere length

**Supplemental Figure I:** The association of telomere length and age in ischemic stroke and control cohorts. The lines represent the linear change in telomere length as a function of age.


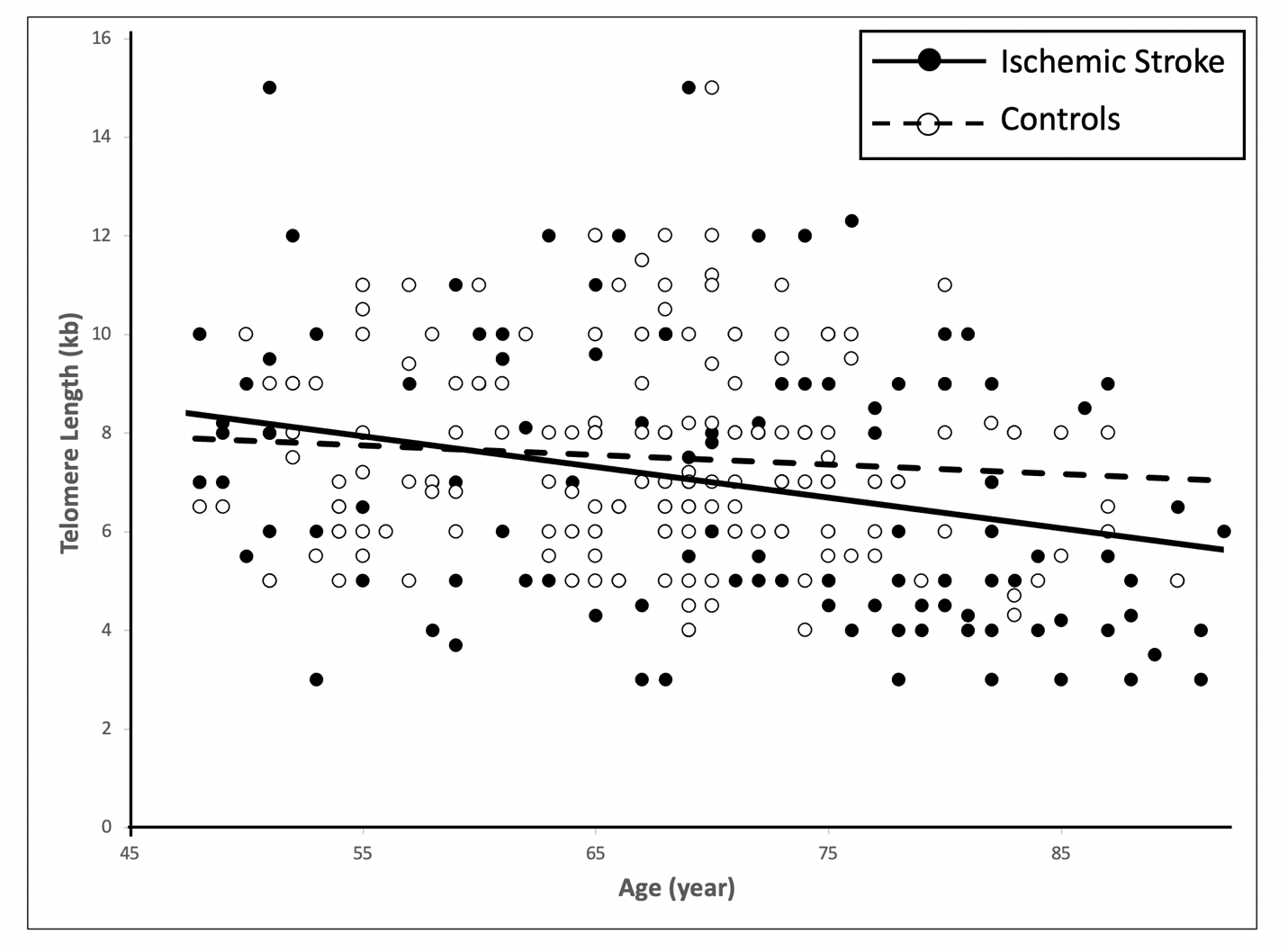

Supplement: Supplementary file 1 — Supplementary Information. [file 41598_2021_90435_MOESM1_ESM.docx]
